# Supplementary material for: The association between maternal body mass index and serial plasma oxytocin levels during labor
Source: PLoS One. 2023 Aug 11;18(8):e0290038. doi: 10.1371/journal.pone.0290038 (PMC10420366; doi:10.1371/journal.pone.0290038)
Supplement: S1 Table — (DOCX) [file pone.0290038.s001.docx]

Table S1. Individual serial levels of plasma OT in the study population (n=40) before and during OT infusion according to BMI.

| Patient Number | BMI | OT levels (ng/mL) before OT infusion started and 20 min after increase of the infusion rate *^a^* | | | | | | | | | | | | |
| --- | --- | --- | --- | --- | --- | --- | --- | --- | --- | --- | --- | --- | --- | --- |
|  |  | **Before** | **3.3** | **6.7** | **10.0** | **13.3** | **16.7** | **20.0** | **23.3** | **26.6** | **30.0** | **33.3** | **36.6** | **40.0** |
| **1** | **22.9** | 32.76 | 38.95 | 54.46 |  |  |  |  |  |  |  |  |  |  |
| **2** | **23.6** | 17.21 | 9.09 | 28.94 | 31.96 | 32.57 |  |  |  |  |  |  |  |  |
| **3** | **25.0** | 1.82 | 4.66 | 21.88 |  |  |  |  |  |  |  |  |  |  |
| **4** | **25.3** | 45.72 | 33.01 | 34.64 | 47.98 | 36.11 | 14.07 |  |  |  |  |  |  |  |
| **5** | **25.7** | 32.07 | 6.20 | 0.27 | 5.54 |  |  |  |  |  |  |  |  |  |
| **6** | **26.8** | 18.60 | 4.47*^b^* |  |  |  |  |  |  |  |  |  |  |  |
| **7** | **27.0** | 15.74 | 6.44 | 18.18 |  |  |  |  |  |  |  |  |  |  |
| **8** | **27.5** | 34.24 | 7.49 | 11.94 |  |  |  |  |  |  |  |  |  |  |
| **9** | **27.9** | 3.52 | 4.90 | 4.33 | 5.53 | 3.47 |  |  |  |  |  |  |  |  |
| **10** | **28.1** | 14.44 | 15.97 | 17.58 | 30.66 |  |  |  |  |  |  |  |  |  |
| **11** | **28.8** | 21.99 | 11.42 | 8.29 | 5.34 | 8.21 *^b^* |  |  |  |  |  |  |  |  |
| **12** | **29.8** | 21.64 | 6.25 | 16.72 |  |  |  |  |  |  |  |  |  |  |
| **13** | **30.1** | 1.20 | 16.04 | 3.13 | 6.95 |  |  |  |  |  |  |  |  |  |
| **14** | **30.1** | 5.34 | 29.48 |  |  |  |  |  |  |  |  |  |  |  |
| **15** | **30.5** | 26.13 | 25.01 | 31.28 | 22.66 | 24.86 | 29.37 | 10.32 |  |  |  |  |  |  |
| **16** | **30.8** | 11.87 | 19.93 | 21.88 |  |  |  |  |  |  |  |  |  |  |
| **17** | **30.8** | 29.70 | 6.92 | 12.07 | 16.21 | 2.81 |  |  |  |  |  |  |  |  |
| **18** | **31.5** | 50.23 | 37.45 | 44.68 | 52.99 |  |  |  |  |  |  |  |  |  |
| **19** | **31.6** | 12.28 | 12.80 *^b^* |  |  |  |  |  |  |  |  |  |  |  |
| **20** | **31.6** | 8.63 | 10.54 | 4.88 |  |  |  |  |  |  |  |  |  |  |
| **21** | **31.6** | 40.50 | 9.01 | 27.63 | 5.65 |  |  |  |  |  |  |  |  |  |
| **22** | **32.0** | 30.69 | 28.63 | 52.27 | 50.20 | 2.87 |  |  |  |  |  |  |  |  |
| **23** | **32.4** | 0.30 | 7.35 | 2.56 | 15.99 |  |  |  |  |  |  |  |  |  |
| **24** | **33.0** | 6.55 | 16.24 | 3.14 | 22.10 |  |  |  |  |  |  |  |  |  |
| **25** | **33.5** | 20.94 | 14.71 | 5.99 | 13.97 |  |  |  |  |  |  |  |  |  |
| **26** | **35.1** | 30.52 | 24.61 | 25.27 | 25.19 | 24.59 | 22.88 | 24.83 | 21.75 | 24.39 | 16.07 | 6.29 |  |  |
| **27** | **35.3** | 6.59 | 25.83 | 1.94 |  |  |  |  |  |  |  |  |  |  |
| **28** | **35.4** | 30.03 | 26.80 | 29.71 | N/A | 40.68 | 42.19 |  |  |  |  |  |  |  |
| **29** | **35.7** | 3.64 | 5.41 | 6.46 | 27.71 | 35.16 | 19.78 | 31.09 | 13.21 |  |  |  |  |  |
| **30** | **36.7** | 12.55 | 18.45 | 6.52 | 18.10 |  |  |  |  |  |  |  |  |  |
| **31** | **37.2** | 43.60 | 31.49 | 33.77 | 29.91 | 28.98 | 29.52 | 21.03 | 28.12 | 27.82 | 19.35 | 15.87 | 25.41 | 23.96 |
| **32** | **37.3** | 27.43 | 10.69 | 21.58 | 37.81 | 31.17 | 35.49 | 20.21 | 14.50 | 15.92 | 27.18 | 2.29 | 29.16 |  |
| **33** | **37.7** | 7.15 | 60.70 | 50.68 | 2.25 | 43.09 | 25.58 |  |  |  |  |  |  |  |
| **34** | **39.2** | 3.28 | 1.36 |  |  |  |  |  |  |  |  |  |  |  |
| **35** | **41.1** | 48.58 | 69.32 | N/A | 51.86 |  |  |  |  |  |  |  |  |  |
| **36** | **42.1** | 51.07 | 46.70 | 16.92 | 38.68 | 65.66 | 69.23 |  |  |  |  |  |  |  |
| **37** | **42.3** | 5.89 | 19.16 | 21.02 | 41.62 | 22.21 | 16.80 |  |  |  |  |  |  |  |
| **38** | **44.2** | 38.70 | 42.42 | 7.13 | 2.05 | 4.68 | 0.18 | 6.26 | 2.21 | 15.45 | 18.64 | 31.47 | 4.73 | 5.58^c^ |
| **39** | **44.3** | 13.05 | 22.55 | 3.52 | 5.42 | 34.29 |  |  |  |  |  |  |  |  |
| **40** | **48.6** | 2.58 | 7.05 | 37.46 | 27.25 | 63.33 | 94.68*^b^* |  |  |  |  |  |  |  |

Oxytocin (OT), BMI, body mass index. N/A, not available. *^a^*Infusion rate = mU/min, increase in rate was made with 3.3 mU/min after 20 minutes if required. *^b^*The sampling was discontinued before the maximal OT infusion was received (drop out or unsuccessful sampling). ^c^Continued sampling was made, at 43.3 mU/min: 36.76 ng/mL at 46.6mU/min: 33.30 ng/mL and at 50.0 mU/min: 24.70 ng/mL.
