# Supplementary material for: The association between maternal body mass index and serial plasma oxytocin levels during labor
Source: PLoS One. 2023 Aug 11;18(8):e0290038. doi: 10.1371/journal.pone.0290038 (PMC10420366; doi:10.1371/journal.pone.0290038)
Supplement: Attachment — Submitted filename: Response to Reviewers.docx [file pone.0290038.s002.docx]

Response to Reviewers

**Additional Editor Comments:**

This study aims to evaluate whether plasma levels of exogenous oxytocin, used for various indications as a uterotonic agent during labor, is affected by BMI. This is an important yet unanswered question. The article is interesting, well written and follows the journal's publishing policies although assessment could not be complete due to the low quality of some of the figures.

Please see reviewer comments below.
*Answer: Thank you*

[Note: HTML markup is below. Please do not edit.]

Reviewers' comments:

Reviewer's Responses to Questions

**Comments to the Author**

1. Is the manuscript technically sound, and do the data support the conclusions?

The manuscript must describe a technically sound piece of scientific research with data that supports the conclusions. Experiments must have been conducted rigorously, with appropriate controls, replication, and sample sizes. The conclusions must be drawn appropriately based on the data presented.

*Reviewer #1: Yes*

*Answer: Thank you*

2. Has the statistical analysis been performed appropriately and rigorously?

*Reviewer #1: Yes*

*Answer: Thank you*

3. Have the authors made all data underlying the findings in their manuscript fully available?

The [PLOS Data policy](https://eur01.safelinks.protection.outlook.com/?url=http%3A%2F%2Fwww.plosone.org%2Fstatic%2Fpolicies.action%23sharing&data=05%7C01%7Cmarie.blomberg%40liu.se%7C0c940f2492ff42a85dfa08db7f27ff4e%7C913f18ec7f264c5fa816784fe9a58edd%7C0%7C0%7C638243582134005249%7CUnknown%7CTWFpbGZsb3d8eyJWIjoiMC4wLjAwMDAiLCJQIjoiV2luMzIiLCJBTiI6Ik1haWwiLCJXVCI6Mn0%3D%7C3000%7C%7C%7C&sdata=gHiUu3On7xnL4pAgyf2emJ6EwZX26i6DrVmTNtFRVJc%3D&reserved=0) requires authors to make all data underlying the findings described in their manuscript fully available without restriction, with rare exception (please refer to the Data Availability Statement in the manuscript PDF file). The data should be provided as part of the manuscript or its supporting information, or deposited to a public repository. For example, in addition to summary statistics, the data points behind means, medians and variance measures should be available. If there are restrictions on publicly sharing data—e.g. participant privacy or use of data from a third party—those must be specified.

Reviewer #1: No

*Answer: The dataset's DOI written in the manuscript should be provided by Dryad (an open data publishing platform) to the journal if they will publish the DOI in a data availability statement in the final published article.*

*This is the temporary link to share the data files with the journal office and reviewers:* <https://datadryad.org/stash/share/47uc8ATlVzTvchC2L8FG5gcZy6QMsK0ekSPU5essYbY>

4. Is the manuscript presented in an intelligible fashion and written in standard English?

PLOS ONE does not copyedit accepted manuscripts, so the language in submitted articles must be clear, correct, and unambiguous. Any typographical or grammatical errors should be corrected at revision, so please note any specific errors here.

Reviewer #1: Yes

*Answer: Thank you*

5. Review Comments to the Author

Please use the space provided to explain your answers to the questions above. You may also include additional comments for the author, including concerns about dual publication, research ethics, or publication ethics. (Please upload your review as an attachment if it exceeds 20,000 characters)

Reviewer #1: I had the pleasure of reviewing the manuscript by Isgren et al. that reports baseline plasma OT concentration in laboring women stratified by weight, and their relative response to standardized OT infusion. The authors report low positive correlation between plasma OT and BMI at baseline, and increased plasma OT in high BMI subjects after OT infusion at 3.3 mU/min, but not at higher dose ranges. I commend the authors for this sample-rich study using a sophisticated technique that is more specific than commercially available OT ELISA assays. I would like the authors to address the following:

1. Aprotinin, a protease inhibitor, was not used during sample collection. It has been recommended to use aprotinin for these assays. The authors can cite this as a limitation.

*Answer: This has been added as a potential limitation in the text, but in order to avoid extra additions (which could lead to an increased variability) we have instead decided to follow a very strict protocol for sampling, storing, sample handling and analysis including the use of a stable isotope labelled internal standard.*

2. None of the figures are high resolution, so I am unable to interpret any of the included figures. I would urge the authors to submit individual figure files for final review as this is the crux of their research.

*Answer: We apologize for the inappropriate quality of the figures. Individual figure files have been uploaded after using PACE to ensure that all figures meet PLOS requirements.*

3. The authors can include a few references for the use of tandem mass spectrometry as a valid assay for OT.

*Answer: We agree, two references have now been added to the manuscript. Hering et al. Approaches to improve the quantitation of oxytocin in human serum by mass spectrometry. Front. Chem. 2022 and Franke et al. Oxytocin analysis from human serum, urine, and saliva by orbitrap LCMS. Drug Test Anal. 2019.*

6. PLOS authors have the option to publish the peer review history of their article ([what does this mean?](https://eur01.safelinks.protection.outlook.com/?url=https%3A%2F%2Fjournals.plos.org%2Fplosone%2Fs%2Feditorial-and-peer-review-process%23loc-peer-review-history&data=05%7C01%7Cmarie.blomberg%40liu.se%7C0c940f2492ff42a85dfa08db7f27ff4e%7C913f18ec7f264c5fa816784fe9a58edd%7C0%7C0%7C638243582134005249%7CUnknown%7CTWFpbGZsb3d8eyJWIjoiMC4wLjAwMDAiLCJQIjoiV2luMzIiLCJBTiI6Ik1haWwiLCJXVCI6Mn0%3D%7C3000%7C%7C%7C&sdata=DpNpzzSbbIpnds1mErL%2B5jDr%2B0pbV%2B5r0iD%2B0UZyW8w%3D&reserved=0)). If published, this will include your full peer review and any attached files.


If you choose “no”, your identity will remain anonymous but your review may still be made public.


**Do you want your identity to be public for this peer review?** For information about this choice, including consent withdrawal, please see our [Privacy Policy](https://eur01.safelinks.protection.outlook.com/?url=https%3A%2F%2Fwww.plos.org%2Fprivacy-policy&data=05%7C01%7Cmarie.blomberg%40liu.se%7C0c940f2492ff42a85dfa08db7f27ff4e%7C913f18ec7f264c5fa816784fe9a58edd%7C0%7C0%7C638243582134005249%7CUnknown%7CTWFpbGZsb3d8eyJWIjoiMC4wLjAwMDAiLCJQIjoiV2luMzIiLCJBTiI6Ik1haWwiLCJXVCI6Mn0%3D%7C3000%7C%7C%7C&sdata=D8tfivKSnVG1rru3A2V7J8sOV3DRwumlznkih1fzwzw%3D&reserved=0).

Reviewer #1: No

While revising your submission, please upload your figure files to the Preflight Analysis and Conversion Engine (PACE) digital diagnostic tool, [https://pacev2.apexcovantage.com/](https://eur01.safelinks.protection.outlook.com/?url=https%3A%2F%2Fpacev2.apexcovantage.com%2F&data=05%7C01%7Cmarie.blomberg%40liu.se%7C0c940f2492ff42a85dfa08db7f27ff4e%7C913f18ec7f264c5fa816784fe9a58edd%7C0%7C0%7C638243582134005249%7CUnknown%7CTWFpbGZsb3d8eyJWIjoiMC4wLjAwMDAiLCJQIjoiV2luMzIiLCJBTiI6Ik1haWwiLCJXVCI6Mn0%3D%7C3000%7C%7C%7C&sdata=J%2FEexHs2aeg3lxWcdzT50vbgA6a0MB55TgGAFw7AalE%3D&reserved=0). PACE helps ensure that figures meet PLOS requirements. To use PACE, you must first register as a user. Registration is free. Then, login and navigate to the UPLOAD tab, where you will find detailed instructions on how to use the tool. If you encounter any issues or have any questions when using PACE, please email PLOS at [figures@plos.org](mailto:figures@plos.org). Please note that Supporting Information files do not need this step.
